# Tuberculosis infection in BCG vaccinated children and adolescents with rheumatological diseases treated by tumor necrosis factor-α inhibitors

**DOI:** 10.1007/s00431-026-07028-9

**Published:** 2026-05-15

**Authors:** Dilber Ademhan Tural, Deniz Doğru, Beste Özsezen, Azer Karaman, Birce Sunman, Halime Nayir Büyükşahin, İsmail Güzelkaş, Didem Alboğa, Meltem Akgül Erdal, Nagehan Emiralioğlu Ordukaya, Yelda Bilginer, Ebru Yalçın, Uğur Özçelik, Seza Özen, Nural Kiper

**Affiliations:** 1 Department of Pediatric Pulmonology, School of Medicine, Ankara Yıldırım Beyazit University, Ankara, Turkey; 2 Department of Pediatric Pulmonology, Ankara Bilkent City Hospital, Ankara, Turkey; 3 Department of Pediatric Pulmonology, School of Medicine, Hacettepe University, Ihsan Dogramaci Children’s Hospital, Ankara, Turkey; 4 Department of Pediatric Pulmonology, School of Medicine, Dokuz Eylul University, Izmir, Turkey; 5 Department of Pediatric Rheumatology, School of Medicine, Hacettepe University, Ihsan Dogramaci Children’s Hospital, Ankara, Turkey

**Keywords:** Children, Adolescents, Tumor necrosis factor-alpha inhibitors, Tuberculosis, Latent tuberculosis infection, BCG

## Abstract

Tumor necrosis factor-alpha (TNF-α) inhibitors increase the risk of developing active tuberculosis (TB) disease through reactivation of latent TB infection (LTBI). We aimed to analyze TB infections in BCG-vaccinated children and adolescents with rheumatologic diseases treated with TNF-α inhibitors in a country of moderate risk for TB. This retrospective study included 261 children with a rheumatic disease who were treated with TNF-α inhibitors and followed up on a fixed schedule between January 2018 and December 2022. Demographic and clinical characteristics, as well as TB screening results, were recorded. The mean age of the patients was 14.0 ± 4.1 years; 56.7% were female. The mean age at initiation of TNF-α inhibitors was 11.0 ± 5.1 years, the mean duration of TNF-α inhibitor use was 4.1 ± 2.7 years, and the mean follow-up time was 4.1 ± 2.6 years. During the study period, 75 (29.0%) patients were diagnosed with LTBI: 44% at the initial evaluation and 56% during follow-up. None of them progressed to TB disease. Only two cases of active TB disease were seen without prior documented LTBI. Age and duration of TNF-α inhibitor use were significantly associated with LTBI positivity.

*Conclusion*: TB screening is necessary in children and adolescents at the initiation of and during TNF-α inhibitor therapy. The risk of LTBI positivity increases with age and longer use of TNF-α inhibitors. No cases progressed from LTBI to active TB, which may support the effectiveness of current screening and prophylaxis strategies in our country.

**Table:** 

**What is Known:** *• TNF-α inhibitors are associated with an increased risk of developing active tuberculosis (TB) disease as a result of the reactivation of latent TB infection (LTBI).* **What is New:** *• Screening for LTBI at initiation and during TNF-α inhibitor treatment, along with appropriate LTBI prophylaxis, prevented LTBI reactivation into active TB disease in BCG-vaccinated patients receiving TNF-α inhibitors.* *• Increasing age and duration of TNF-α inhibitor treatment are associated with the risk of LTBI positivity.*

**What is Known:**

*• TNF-α inhibitors are associated with an increased risk of developing active tuberculosis (TB) disease as a result of the reactivation of latent TB infection (LTBI).*

**What is New:**

*• Screening for LTBI at initiation and during TNF-α inhibitor treatment, along with appropriate LTBI prophylaxis, prevented LTBI reactivation into active TB disease in BCG-vaccinated patients receiving TNF-α inhibitors.*

*• Increasing age and duration of TNF-α inhibitor treatment are associated with the risk of LTBI positivity.*

## Introduction

Tumor necrosis factor-alpha (TNF-α) plays a significant role in the pathogenesis of various systemic diseases, including childhood rheumatological disorders. TNF-α inhibitors have improved the clinical outcomes and prognosis of the diseases. TNF-α regulates the host immune response to infection with *Mycobacterium tuberculosis* and activates the formation of granulomas to prevent tuberculosis (TB) dissemination [1].

Tuberculosis (TB), despite advances in diagnosis and treatment, remains among the most critical public health problems in the world [2]. It is estimated that 10.6 million people developed active TB disease worldwide in 2022, including approximately 1.3 million children under the age of 15 years [3]. *Mycobacterium tuberculosis* exposure may result in active TB disease or latent TB infection (LTBI). LTBI is a persistent immune response to stimulation by *Mycobacterium tuberculosis* antigens without evidence of active TB’s clinical and radiological characteristics. Non-treated LTBI has a 5–10% lifetime risk of progressing to TB disease [4]. The risk may vary depending on the host’s immune status and TB burden. While the TB burden in a given region mainly affects the risk of exposure to *Mycobacterium tuberculosis*, TNF-α inhibitors are associated with an increased risk of developing active TB disease due to LTBI reactivation, as they impair host immune responses essential for controlling *Mycobacterium tuberculosis* [5–13]. Turkiye is a country where the prevalence of TB is at a moderate level [3, 14].

This increased risk necessitates screening for TB infections (active TB disease or LTBI) before and during TNF-α inhibitor treatment [15–17]. Screening for LTBI has been reported to significantly reduce the risk of TB disease with the use of prophylaxis for LTBI in patients receiving TNF-α inhibitors [15–18]. Tuberculin skin test (TST) and interferon-gamma release assay (IGRA) are commonly used to diagnose LTBI, which reflects the immune response (memory T cells) to exposure to *Mycobacterium tuberculosis* [19]. However, both methods have some limitations in childhood. TST interpretation is challenging in BCG-vaccinated individuals, as cross-reactivity with BCG and non-tuberculous mycobacteria may yield false-positive results, potentially leading to an overestimation of true LTBI prevalence. Conversely, false-negative TST results may occur in immunocompromised patients due to impaired tuberculin reactivity. IGRA may not be sensitive enough in children younger than five years old [20]. International guidelines provide different recommendations for the use of TST and IGRA [21–23].

The TB risk and the best TB screening method are not well known precisely in BCG-vaccinated children treated with TNF-α inhibitors. We used the term “TB infections” to refer to both active TB disease and LTBI in our study. We aimed to determine the frequency of TB infections among BCG-vaccinated children and adolescents with rheumatologic diseases treated with TNF-α inhibitors, and to critically evaluate the effectiveness and safety of our TB screening program based on real-world experience.

## Method

### Study design and participants

This retrospective study included patients aged 18 years or younger with rheumatological disease who were treated with TNF-α inhibitors and followed in the pediatric pulmonology department of a single tertiary care center between January 2018 and December 2022.

Written consent from each patient and their parent was obtained before initiating the TNF-α inhibitors. However, informed patient consent for the study was waived because it is a retrospective study. The study received approval from the Institutional Research and Ethics Committee of Hacettepe University (KA-23019) and was conducted in accordance with the Helsinki Declaration on Human Rights.

### Data collection and definitions

The demographic and clinical characteristics of the patients were obtained from their medical records, including age, sex, BCG vaccination status, type of rheumatological disease, duration of the disease, types of TNF-α inhibitors, duration of TNF-α inhibitors treatment, other used treatments, history of TB, contact with patients who had TB diseases, TST and IGRA results, and history of LTBI prophylaxis.

Our country’s routine childhood immunization program administers a single dose of the BCG vaccine to babies at two months of age.

LTBI was defined as any positive screening test result (TST and/or IGRA) without clinical or radiological signs of TB disease [21]. Active TB disease diagnosis was based on clinical, radiological, and/or microbiological findings [21]. The term “TB infection” refers to both active TB disease and LTBI in our study. LTBI prophylaxis was given as isoniazid for 9 months or rifampicin for 4 months [24].

The patients’ childhood rheumatological diseases were categorized into three groups: juvenile idiopathic arthritis (JIA), autoinflammatory diseases (AID), and vasculitis.

JIA consisted of systemic JIA, polyarticular JIA, oligoarticular JIA, enthesitis-related arthritis, psoriatic arthritis, and undifferentiated arthritis.

AIDs included patients with familial Mediterranean fever, chronic nonbacterial osteomyelitis, cryopyrin-associated periodic syndrome, and tumor necrosis factor receptor-associated periodic syndrome.

Patients with vasculitis included those with Behçet disease, Takayasu arteritis, and adenosine deaminase-2 deficiency.

### Tuberculosis infection screening procedure

**Fig. 1:**
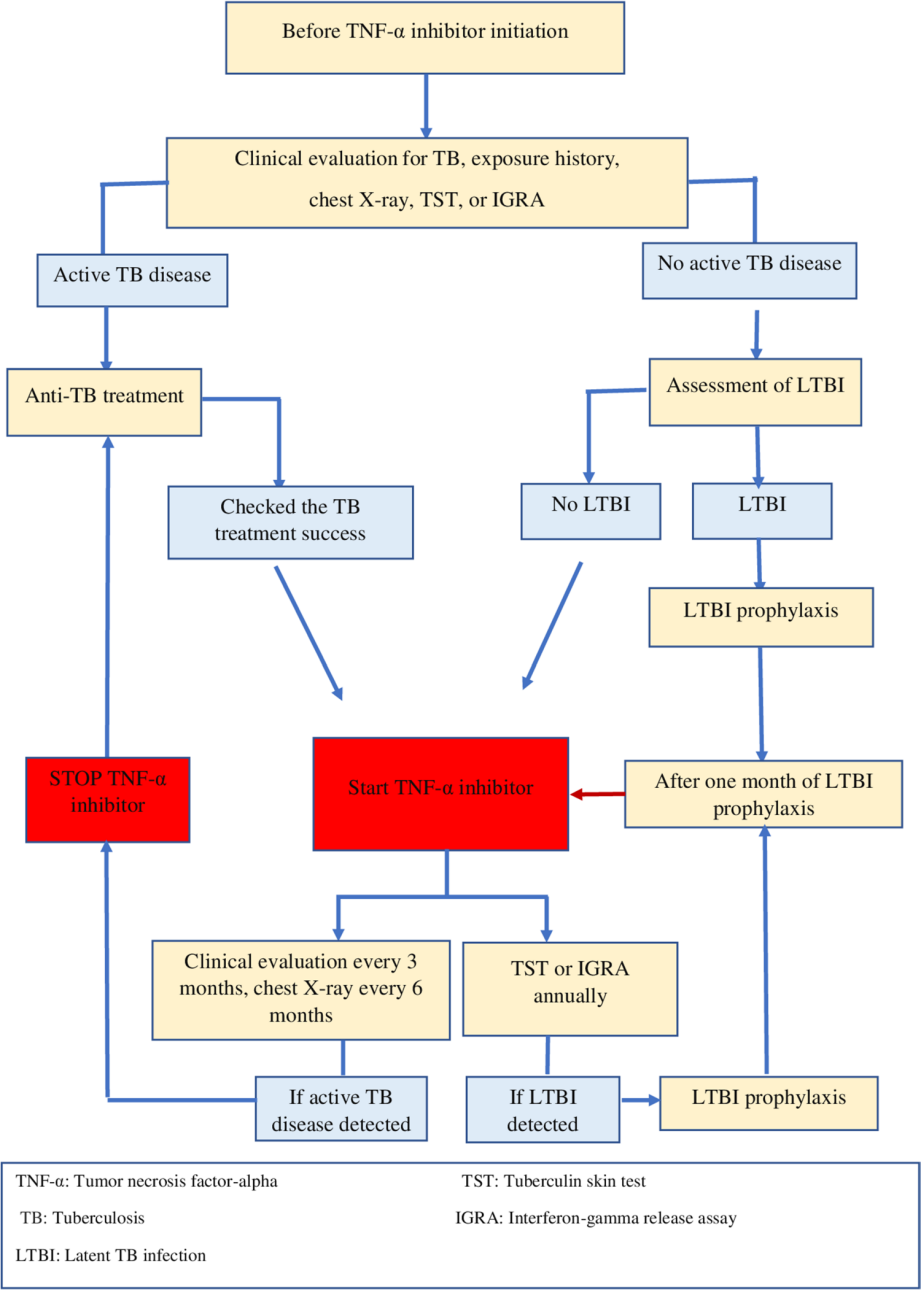
The summary of the tuberculosis infection screening protocol

All patients were screened for TB infection before starting TNF-α inhibitors and during follow-up. Patients were clinically evaluated every 3 months, and chest radiographs were performed every 6 months. TST and/or IGRA were obtained every 12 months. The TB infection screening procedure was summarized in Fig. 1. IGRA screening could not be performed in all patients; results were available only for a subset of patients at baseline and during TNF-α inhibitor therapy.

If there was any suspicion of active TB disease, sputum or early-morning gastric aspirates were obtained, and flexible bronchoscopy for acid-fast bacilli (AFB), TB polymerase chain reaction (PCR), and TB culture were performed, if indicated. Chest tomography and family TB screening were also obtained.

The Mantoux method was used for the TST test. A standard dose of five tuberculin units of purified protein derivative (0.1 mL) was applied intradermally into the forearm and evaluated after 48 to 72 h. TST size was measured in millimeters to assess transverse induration.

IGRA was performed by QuantiFERON®-TB Gold Plus (QFT-Plus) test. Four milliliters of peripheral venous blood was taken from each patient, and 1 ml was added to each of the heparinized four tubes in the QFT-Plus (Nil, TB1, TB2, and mitogen tubes). The tubes were immediately turned upside down at least ten times to ensure that the entire inner surface of the tube was covered with blood. The tubes were first incubated at 37 °C for 16–24 h as soon as possible after the collection. The longer the incubation is delayed, the greater the possibility of an indeterminate test result. After incubation, the tubes should be centrifuged at 2000 g for 15 min to separate the plasma. The enzyme immunoassay (EIA) method was used for the analyses.

### Evaluation of LTBI screening

1. The initial evaluation, according to TST and IGRA results [24];
  BCG-vaccinated children with a reaction TST size ≥ 10 mm or BCG-unvaccinated children with a reaction TST size ≥ 5 mm or regardless of BCG status, who received 15 mg/day systemic steroid treatment for more than 2 weeks in the last 3 months, with TST size ≥ 5 mm or IGRA positivity, were defined as LTBI after excluding TB disease. In patients with LTBI, TNF-α inhibitor treatment was usually given after 1 month of LTBI prophylaxis.
2. During the follow-up, patients without LTBI [24] were monitored;
  TST conversion is defined as an annual increase in TST induration of > 5 mm. TST conversion was also considered LTBI after excluding TB disease, in addition to the initial evaluation criteria of TST and IGRA results.
3. During the follow-up, patients with LTBI [24];
  Patients with LTBI were followed up clinically and with chest radiographs when needed.

### Statistics

Statistical analyses were performed using SPSS 28.0 (IBM, SPSS, Chicago, IL, USA). Findings from descriptive analyses are reported as relative and absolute frequencies for categorical variables and as mean (standard deviation (SD)) for quantitative variables. In all statistical tests, *p* < 0.05 was considered statistically significant. Multivariable logistic regression analyses were performed to identify risk factors associated with LTBI positivity detected during screening. Variables with a *p*-value ≤ 0.20 in univariate analyses were included in the multivariable model. Multivariate logistic regression analysis was performed using the Enter method. Odds ratios (ORs) with 95% confidence intervals (CIs) were calculated.

## Results

### Patient characteristics

The study included 261 patients. The mean age of the patients was 14.0 ± 4.1 years; 148 (56.7%) were females, and all were BCG vaccinated. Of the patients, 69.0% were followed up for JIA, 10.7% for AID, 9.2% for vasculitis, and 11.1% for both JIA and AID. Among all patients, 69.7% received etanercept, 28.4% received adalimumab, and 1.9% received infliximab. The mean age at which TNF-α inhibitors were started was 11.0 ± 5.1 years, and the mean duration of TNF-α inhibitor use was 4.1 ± 2.7 years. The mean of the follow-up time at our clinic was 4.1 ± 2.6 years. The demographic and clinical characteristics of the study population are summarized in Table 1. Almost 90.0% of patients (*n*, 242, 92.7%) had previously or were currently receiving one or more immunosuppressive therapies (methylprednisolone, methotrexate, azathioprine, cyclosporine) before the TNF-α inhibitors were started.

**Table 1:** Demographic and clinical characteristics of the study participants

Age in years, mean (standard deviation)	14.1 ($\pm$4.1)
Sex, females *n* (%)	148 (56.7)
Age of the onset TNF-α inhibitors in years, mean (standard deviation)	11.0 (± 5.1)
Duration of TNF-α inhibitors in years, mean (standard deviation)	4.1 (± 2.7)
The follow-up time in years, mean (standard deviation)	4.1 (± 2.6)
Rheumatological diseases *n* (%)	
Juvenile idiopathic arthritis (JIA) Autoinflammatory disease (AID) Vasculitis Both JIA and AID	180 (69.0) 28 (10.7) 24 (9.2) 29 (11.1)
The use of TNF-α inhibitors *n* (%)	
Etanercept Adalimumab Infliximab	182 (69.7) 74 (28.4) 5 (1.9)

All patients were evaluated for “TB infection,” which included both active TB disease and LTBI, at the initial evaluation and during TNF-α inhibitor treatment.

### Latent tuberculosis infection

All 261 patients had at least one TST measurement, and 75.0% (*n* = 196) had at least one IGRA test. IGRA testing was not available to all patients because it was not covered by the national reimbursement system during the study period and required out-of-pocket payment.

**Fig. 2:**
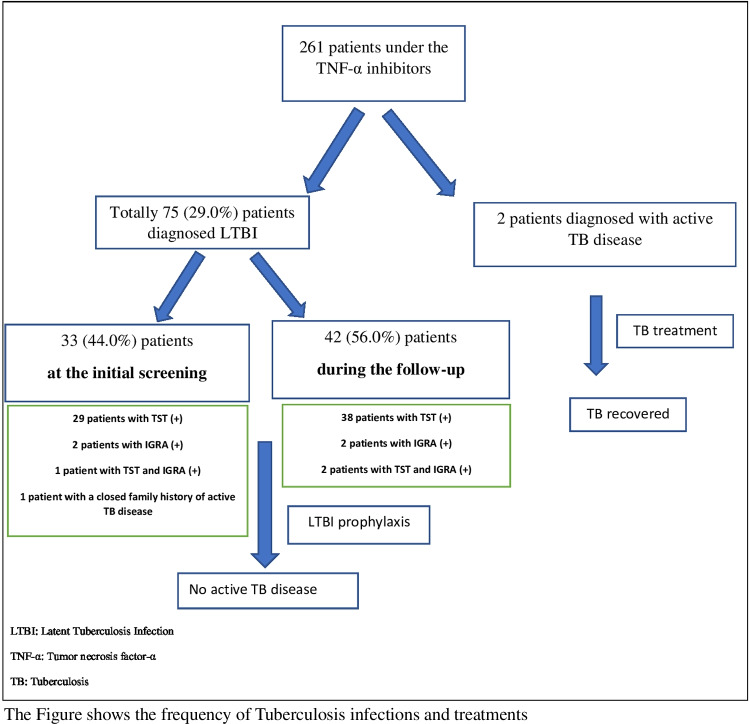
The summary of tuberculosis infections in the study population

In total,75 (29.0%) patients were diagnosed with LTBI during the study period. Of these patients, one (1.3%) was diagnosed with LTBI due to a family history of TB before using TNF-α inhibitors, 32 (42.7%) were diagnosed at the initial screening, and 42 (56.0%) patients were diagnosed during the follow-up (Fig. 2). LTBI was detected in 51 patients with JIA, 11 with AID, 10 with both JIA and AID, and only 3 patients with Vasculitis in our cohort.

At the initial evaluation, all patients were evaluated with TST and chest X-ray, and 66.0% (*n* = 173) also had IGRA. LTBI was defined as TST positivity in 29 patients, IGRA positivity in 2 patients, TST and IGRA positivity in 1 patient, and a history of close contact with a patient with TB disease. TNF-α inhibitor treatment was initiated 1 month after LTBI prophylaxis.

During the study period, 42 (56.0%) patients were diagnosed with LTBI while receiving TNF-α-inhibitor treatment. In these patients, LTBI was defined as TST positivity in 38 patients, IGRA positivity in 2 patients, and as both TST and IGRA positivity in 2 patients. TNF-α-inhibitor treatment was continued after 1 month of LTBI prophylaxis.

None of the patients with LTBI had respiratory symptoms or chest radiographic findings consistent with TB disease. Patients with both TST and IGRA positivity were also evaluated with chest tomography, and no specific findings of TB disease were noted.

Except for one, patients diagnosed with LTBI were given at least 9 months of isoniazid prophylaxis. One patient took rifampicin for 4 months because of the liver toxicity of isoniazid.

None of the patients diagnosed with LTBI developed active TB disease during the follow-up.

### Active tuberculosis disease

Only two, among 261 patients, both female, were diagnosed with active TB disease during the study period.

A 16-year-old girl followed up with JIA and has been using etanercept for 2 years. She had no LTBI both at the beginning and during the TNF-α inhibitor treatment. During the third year of TB screening, she had a cough for more than 1 month despite receiving nonspecific antibiotic treatment (clarithromycin). Both TST and IGRA were found. The chest tomography showed mediastinal and bilateral hilar lymphadenopathies bigger than 1 cm and a tree-in-bud appearance (multiple areas of centrilobular micronodules with a linear branching pattern). Family TB screening, gastric aspirate AFB, TB PCR, and TB culture were negative. TB disease was diagnosed based on clinical and radiological findings. After completing TB treatment (2 months of isoniazid, rifampicin, ethambutol, and pyrazinamide; 4 months of isoniazid and rifampicin), she continued to receive etanercept without any complications for more than two years of follow-up.

The other patient, a 16.5-year-old girl diagnosed with Takayasu arteritis, has been taking adalimumab for more than 6 years and had no previously detected LTBI. She had a cough at her last visit. The chest x-ray showed right-sided pleural effusion. Both TST and IGRA positivity were found. Pleural fluid and bronchoalveolar lavage were AFB-negative, TB PCR-positive, and yielded *Mycobacterium tuberculosis complex* growth. She is still taking TB treatment, and adalimumab has been discontinued.

### Risk factors of latent tuberculosis infection in patients receiving TNF-α inhibitors

Logistic regression analyses were performed to identify risk factors for LTBI positivity detected by follow-up screening. The analysis was restricted to the 228 patients without LTBI at initial evaluation, among whom 42 developed LTBI during follow-up and were considered outcome events. The following variables were first evaluated using univariate logistic regression: age, sex, age at diagnosis, type of rheumatological disease, type of TNF-α inhibitor, age at the start of TNF-α inhibitor treatment, duration of TNF-α inhibitor therapy, and receipt of previous or current immunosuppressive therapies other than TNF-α inhibitors. Variables with a *p*-value < 0.20 were included in the multivariable logistic regression model. The final multivariable model included age, rheumatological disease type, TNF-α inhibitor type, and duration of TNF-α inhibitor therapy.

In the multivariable analysis, age (OR, 1.098 per 1-year increase; 95% CI, 1.019–1.184; *p* = 0.015) and duration of TNF-α inhibitor therapy (OR, 3.547 per 1-year increase; 95% CI, 1.774–4.680; *p* = 0.012) were positively associated with LTBI positivity, whereas a diagnosis of vasculitis was inversely associated with LTBI positivity compared to other rheumatological diagnoses (OR, 0.221; 95% CI, 0.051–0.961; *p* = 0.044) (Table 2). Notably, each additional year of TNF-α inhibitor therapy was associated with a 3.5-fold increase in the odds of LTBI positivity, representing a substantial effect size. Model fit was assessed using the Hosmer–Lemeshow goodness-of-fit test (*p* = 0.370), and variance inflation factors (VIFs < 5) confirmed the absence of significant multicollinearity. No statistically significant interaction between age and duration of TNF-α inhibitor therapy was observed.

**Table 2:** Risk factors for having LTBI

	B	OR	% 95 CI	*p* value
Age	0.094	1.098	1.019–1.184	0.015
Duration of TNF-α inhibitor use	0.101	3.547	1.774–4.680	0.012
Having vasculitis	− 1.512	0.221	0.051–0.961	0.044

*CI* confidence interval, *B* regression coefficient, *OR* odds ratio

The risk of LTBI increases with age, duration of TNF-α inhibitor use, and having a rheumatologic disease other than vasculitis. Other factors, such as sex, type of TNF-α inhibitor, age at the start of TNF-α inhibitor treatment, and receipt of previous or current immunosuppressive therapies, did not show a significant additional risk of LTBI.

## Discussion

Our study demonstrates that screening for TB infection, both LTBI and active TB disease, is necessary in children and adolescents at the initiation of TNF-α inhibitor therapy and throughout therapy. Increasing age and duration of TNF-α inhibitor treatment are associated with the risk of LTBI positivity. No one developed TB disease after LTBI treatment, which may suggest that LTBI prophylaxis prevents progression to TB disease. The results may support the effectiveness and sufficiency of the screening protocol used in our country.

Receiving TNF-α inhibitors is linked to a higher risk of developing TB infections, including LTBI and active TB disease [25]. Nearly 30% of the study participants were diagnosed with LTBI during the study period, a rate similar to that found in previous studies from our country on children using TNF-α inhibitors [25–28]. Bayramoğlu et al. [25] detected more than half of the LTBI cases during initial screening, while we detected less than half at that stage. More than 50% of LTBI cases are detected during TNF-α inhibitor treatment, and we found that the risk of LTBI positivity increases with age and with longer TNF-α inhibitor use. These findings emphasize the importance of screening for LTBI in children and adolescents with rheumatological diseases, both at the initial screening and throughout TNF-α inhibitor treatment. However, the optimal strategy for repeat LTBI screening during TNF-α inhibitor therapy remains debated and appears to depend largely on the regional epidemiology of tuberculosis. While low-incidence TB countries often recommend repeat testing only in the presence of new exposure or clinical suspicion [29], a different approach may be warranted in regions with intermediate or high TB burden [30]. In such settings, ongoing community transmission increases the likelihood of newly acquiring *Mycobacterium tuberculosis* infection during long-term TNF-α inhibitor treatment. Therefore, periodic LTBI screening during TNF-α inhibitor treatment has been recommended to detect new infections early and prevent progression to active TB disease. Countries with intermediate TB incidence, like Turkiye, may especially benefit from ongoing surveillance, as patients on TNF-α inhibitors remain vulnerable not only to reactivation of previously undetected LTBI but also to newly acquired infections during treatment. Our findings, which show that a significant portion of LTBI cases were identified during follow-up rather than at initial screening, may support the importance of continuous monitoring in similar epidemiological settings.

In our cohort, LTBI was more common in patients with JIA than in those with AID or vasculitis. This difference may be related to the larger number of JIA patients in the study cohort and to the longer cumulative duration of immunosuppressive and TNF-α inhibitor therapies typically used in JIA management. However, because subgroup sizes were limited, especially in the vasculitis group, these findings should be interpreted cautiously. Future studies using larger, disease-specific cohorts are needed to clarify whether LTBI prevalence genuinely differs across rheumatologic indications or primarily reflects cohort composition. In our multivariate analysis, vasculitis was associated with a lower risk of LTBI positivity; however, this result should be interpreted with caution. The small number of patients with vasculitis in our cohort (representing only 4% of LTBI-positive cases) suggests that the analysis may be underpowered and susceptible to residual confounding. Therefore, this observation should be considered hypothesis-generating rather than indicative of a true protective effect. Further large-scale prospective studies are needed to better clarify the relationship between underlying rheumatic disease subtypes and the risk of LTBI during TNF-α inhibitor treatment. In the multivariable analysis, age and duration of TNF-α inhibitor therapy were associated with a higher risk of LTBI positivity. The effect size for TNF-α inhibitor duration was particularly large (OR, 3.547 per 1-year increase), with a wide confidence interval (95% CI, 1.774–4.680). This finding should be interpreted with caution, given the small sample size and the study’s retrospective design.

When LTBI is not diagnosed and properly treated, it may progress to active TB disease in 5–10% of cases [4]. None of our patients diagnosed with LTBI developed active TB disease during the 4-year mean follow-up period. Several previous studies from our country have also reported no progression from LTBI to active TB disease [17, 25, 27, 31]. Kilinc et al. reported that only two patients progressed from LTBI to active TB disease despite the LTBI prophylaxis [26]. These studies from our country on children using TNF-α inhibitors and our results indicate that the progression rate from LTBI to active TB disease is quite low. Although no LTBI cases progressed to active TB disease during follow-up, this finding should be interpreted with caution, considering the relatively small sample size and limited follow-up duration. Therefore, our results may support rather than definitively prove the potential effectiveness of the screening and prophylaxis strategy.

TST and IGRA can be affected by various conditions, particularly in children with chronic diseases; neither test serves as a gold standard for diagnosing LTBI [32, 33]. Most recent studies and guidelines recommend a dual-screening approach combining TST and IGRA in this patient population [34–37]. In our cohort, most LTBI diagnoses were based on TST positivity, whereas IGRA positivity was relatively infrequent. The subgroup of patients who underwent IGRA testing may not fully represent the entire cohort, so the relative performance of each test should be interpreted with this limitation in mind. Interpreting TST results requires particular caution in our population because BCG vaccination is universally administered in Turkiye. In BCG-vaccinated individuals, TST false-positivity rates have been reported to range from 20 to 40%, depending on the number of BCG doses, time since vaccination, and the TST cut-off used [40, 41]. Accordingly, our LTBI rate (29%) should be interpreted cautiously, as it may reflect a combination of true infection and false-positive TST results. However, several factors may attenuate the contribution of false positives in our cohort. The effect of BCG on TST reactivity declines with increasing age and time since vaccination [21, 38–40], and BCG-related TST reactivity is substantially more persistent when vaccination occurs after infancy [41]. This suggests that the false-positive burden may be towards the lower end in our universally infant-vaccinated population. Moreover, larger induration sizes are more likely to reflect true *Mycobacterium tuberculosis* infection rather than a vaccine response [40], and cross-reactivity with non-tuberculous *mycobacteria* is generally limited when appropriate cut-off values and clinical risk assessment are used. IGRA may offer greater specificity in BCG-vaccinated populations, as it uses antigens absent in BCG strains and in most environmental mycobacteria [7, 8]. Nevertheless, TST remains widely used due to its accessibility, low cost, and extensive clinical experience, particularly in pediatric settings. Therefore, TST results should always be interpreted in conjunction with epidemiological risk factors and clinical context. Taken together, these considerations may partly explain the discordance between frequent TST positivity and relatively low IGRA positivity observed in our cohort, and underscore the importance of integrating IGRA results whenever feasible.

Two of the 261 study participants (0.8%) developed active TB disease during a mean follow-up of more than four years. Notably, both cases occurred in patients without prior documented LTBI, a finding with important clinical implications that warrants careful consideration. Two mechanistic explanations should be considered. First, these cases may represent false-negative screening results, as the sensitivity of both TST and IGRA is known to be reduced by immunosuppression, potentially masking true LTBI at the time of initial screening [40, 41]. Second, de novo acquisition of *Mycobacterium tuberculosis* during the follow-up period cannot be excluded, particularly in an intermediate TB-burden setting such as Turkiye. Distinguishing between these two possibilities is inherently difficult in clinical practice; however, each scenario has distinct implications for the screening strategy. If false-negative results are the predominant mechanism, this underscores the inadequacy of a single pre-treatment screening episode for children receiving TNF-α inhibitor therapy and supports the use of combined TST and IGRA testing to maximize sensitivity. If a new infection during follow-up is the primary driver, this highlights the critical importance of repeated TB infection screening at regular intervals throughout TNF-α inhibitor therapy, not only at baseline. In either case, a negative screening result should never be interpreted as conferring complete protection against future active TB disease in this population. These findings are consistent with previously reported rates in similar cohorts. Kilic et al. [27] reported active TB disease in 1 of 144 children (0.7%); Barut et al. [42] identified de novo active TB disease in 1 of 234 children (0.4%); and Kılınç et al. [26] reported 2 cases of LTBI reactivation progressing to active TB disease. A recent Spanish study, conducted in a low TB-burden setting, identified active TB disease in only 0.7% of TNF-α inhibitor-treated children over a median follow-up exceeding six years [29]. Collectively, these data suggest that progression to active TB disease is rare in well-screened cohorts, and may reflect the overall effectiveness of current LTBI diagnostic and treatment protocols. Nevertheless, the occurrence of active TB disease in patients without prior LTBI detection highlights a residual risk that cannot be eliminated by baseline screening alone. We therefore propose that, in intermediate TB-burden settings, periodic rescreening during TNF-α inhibitor therapy, particularly following new TB exposure, clinical deterioration, or intensification of immunosuppression, should be considered as part of routine clinical management. While the optimal screening interval remains to be established by prospective studies, our findings support a policy of ongoing clinical vigilance rather than a single pre-treatment assessment. It should be acknowledged that the applicability of these recommendations may be limited in low TB-burden regions, where the absolute risk of new infection during follow-up is substantially lower. Therefore, screening frequency and intensity should ultimately be guided by regional TB prevalence, individual patient risk factors, and local clinical guidelines.

The study has several limitations. First, it is a single-center study, which limits the broader generalization of the results. Second, there was no control group; therefore, we could not compare the frequency of LTBI and active TB disease between patients who used TNF-α inhibitors and those who did not. Third, IGRA testing could not be performed for all patients due to reimbursement restrictions. As a result, only a subset of patients underwent IGRA screening, potentially introducing selection bias if the tested patients differed systematically from the untested. This should be considered a source of selection bias, not merely a logistical constraint, when interpreting the study’s LTBI prevalence estimates and their generalizability. Fourth, we did not assess the relative importance or agreement of the TST and IGRA in diagnosing LTBI. Because only a small proportion of LTBI cases were IGRA-confirmed, subgroup analyses comparing TST-only and IGRA-confirmed cases were not statistically reliable. This limitation should be considered when interpreting the robustness of LTBI classification in our cohort. Finally, the study has all the limitations typical of a retrospective study. Despite these limitations, our study was conducted at a tertiary hospital, where patients came from many different regions of the country. Our results demonstrate the effects of long-term TNF-α inhibitor therapy in BCG-vaccinated children with rheumatologic diseases, based on real-world experience.

In conclusion, the study demonstrated that there is no reactivation of LTBI to active TB disease in pediatric rheumatic patients using TNF-α inhibitors, which may be attributable to screening and effective preventive therapy at LTBI initiation and during TNF-α inhibitor treatment. Importantly, negative LTBI screening results do not fully exclude the risk of future active TB disease in children receiving TNF-α inhibitors; therefore, ongoing clinical vigilance is warranted throughout TNF-α inhibitor use, irrespective of screening outcomes. The results may indicate that the screening protocol used in our country could be effective in similar epidemiologic settings; however, further multicenter studies are necessary to confirm its generalizability.

## Data Availability

The datasets used in the present study are available from the corresponding author upon reasonable request.

## Abbreviations

TNF-α: Tumor necrosis factor-alpha
TB: Tuberculosis
LTBI: Latent TB infection
BCG: Bacillus Calmette‐Guerin
TST: Tuberculin skin test
IGRA: Interferon-gamma release assay
JIA: Juvenile idiopathic arthritis
AID: Autoinflammatory disease
AFB: Acid-fast bacilli
PCR: Polymerase chain reaction
